# Physical Attributes of Housing and Elderly Health: A New Dynamic Perspective

**DOI:** 10.3390/ijerph16244961

**Published:** 2019-12-06

**Authors:** Zan Yang, Yuqi Fu

**Affiliations:** Hang Lung Center for Real Estate, Department of Construction Management, Tsinghua University, Beijing 100084, China; fuyq19@mails.tsinghua.edu.cn

**Keywords:** elderly health, housing, medical expenditures, China

## Abstract

Maintaining health and improving the quality of life of the elderly is extremely challenging in an aging society. In this study, the relationship between housing and the independence and functional capabilities of the elderly is examined, and the effect of housing conditions on health improvements and their economic benefits for the elderly in terms of medical expenditures are assessed. The study is based on the Chinese Health and Retirement Longitudinal Study (CHARLS), which was conducted in 2011 and 2013. Two indices that measure housing conditions and the health status of the elderly were run through regression and state-transition models. Housing was found to have a positive relationship with the health of the elderly, and the improvement of housing conditions could significantly change health status and decrease medical expenditures. The importance of maintaining the health of the elderly through housing adaptations and the economic benefits of housing interventions are highlighted, as these can contribute to both public health and housing adaption subsidy policies.

## 1. Introduction

### 1.1. Background

Housing can significantly affect the health of the elderly, as in its broader sense, housing is not simply shelter but also valuable wealth, and it is socially linked to individuals’ wellbeing. The daily activities of the elderly are also predominantly conducted in the home, and people may have a strong emotional attachment to the house in which they have lived for many years [1]. Housing also serves as a platform for achieving a favorable living environment and service outcomes, and it thus can improve the independence and health of the elderly [2]. In 2017, it was estimated that 962 million people were aged 60 or above worldwide, comprising 13% of the global population: by 2050, over 30% of the global population, excluding Africa, will be elderly (aged 60+) [3]. The population of those aged 60 or above is growing by 3% per year, which is faster than all younger age groups [3]. Thus, maintaining the health of the elderly and improving their quality of life is an increasingly challenging task. Existing studies have investigated these problems from several different aspects, such as universal design [4], aging in place [5], and person–environment fit [6], and have incorporated the role of innovation in new technologies [7]. Understanding the relationship between housing and the health of the elderly is helpful in addressing this important issue. This issue should be of primary importance in public health policies that address the issue of aging in place.

The relationship between housing and the health of the elderly is complex. Its correlation goes far beyond assessing health-related physical hazards and accident prevention, and it involves the interaction between elderly competence and housing [8]. Housing that is beneficial to health not only increases the safety and comfort of the dwelling but also improves various living and utility functions in the daily life of the aging [1]. Most of the extensive studies related to housing and its health impacts on older people have focused on the effects of housing on falls [9], mobility [5], or mental health [10]: few have provided overall assessments of functional capacity [2]. The need for further investigations into the elderly’s activities of daily living (ADLs) (going beyond examining just walking and mobility) has been highlighted [9]. This study fills these research gaps by examining the impact of housing on elderly independent living, as measured by the full spectrum of ADL functionality and the instrumental activities of daily living (IADLs), which capture the role that housing can play in the levels of independence and functional capabilities of the elderly.

In addition, in this paper, the impact of housing conditions on health changes in the elderly is measured, and the economic value of housing on health is further assessed. As the numbers of the elderly have increased in many countries, so have their healthcare expenses, leading to extensive research on the health, well-being, and life expectancy of an increasingly elderly population [4,11,12]. To our knowledge, this is the first study that has aimed to capture the dynamic link between housing and changes in the health of the elderly, as well as the economic benefits of housing improvement on their health. There are always theoretical and empirical challenges in finding concrete evidence for the impact of housing on health from a dynamic perspective because of the complex interconnectedness between housing, social and economic conditions, the health of the elderly [13], the heterogeneous competence of elderly [2], and the difficulty of measuring the degree of housing improvements. Few quantitative studies have addressed the impact and economic benefits of housing improvements. In this study, the threshold levels of different health statuses were estimated based on two-year panel data using a state-transition model. By comparing the different levels of health status development under the different housing conditions over the two years, we were able to capture the effects that housing conditions have on improving health and thus gain insight into the benefits of housing adaptations on the health of the elderly. In addition, the potential economic benefits of housing improvements were assessed by measuring the correlation between health and medical expenditures. Thus, this study provides theoretical and empirical contributions to developing and improving housing interventions and identifying their economic benefits. It also contributes to the formation of policies on housing adaptation planning and subsidies.

In this study, housing conditions were found to be positively correlated with the health status of those between 60 and 80 years of age (according to Chinese Health and Retirement Longitudinal Study (CHARLS) data from 2011 and 2013). Moreover, it was indicated that housing improvements benefit the health improvement of the elderly and that housing conditions have a significant impact on curbing medical expenditures among the elderly, particularly among younger and healthier old people.

### 1.2. Literature Review

The competence–environment stress model proposed by Lawton and Nahemow [8] provides a general conceptual foundation for the relationship between the environment and people. In housing and elderly health studies, the importance of this model lies in its emphasis on the role of the housing environment and the competencies of individuals to support or undermine individual functions in elderly health, which constitute the mechanisms involved in the links between housing and elderly health [14]. From this perspective, the physical hazards of housing and the elderly’s interactions with the housing environment are both important to understanding the relationship between housing and the health of the elderly.

There is a great deal of research on housing and elderly health in terms of the indirect economic aspects of housing, including housing ownership [15,16], affordability [17], housing wealth [18], and the direct physical hazards of housing [19] due to services and resources [20,21]. In terms of the effects of the physical characteristics of housing on the health of the elderly, the concept of the “healthy housing environment” [22] has been applied, which identifies the attributes of safety, health, amenities, and convenience. Many studies have focused on single dimensions of housing [23], such as barrier-free facilities [24], housing typology [25], lighting [26], noise [27,28], the state of home disrepair [29], access to residential facilities [30], and access to housing and rooms [29]. However, the effects of combined housing attributes on health have been neglected in the literature [23]. More research is needed to explore the effects of housing as a whole on health [23] or to investigate the relationship between perceived housing and health [19].

In addition, the effects of housing improvements on health [31], which could be of value to policymakers and housing providers, have rarely been examined. The current research has been focused on the influence of housing improvements on psychological well-being, levels of satisfaction [32], and quality of life [1]. Further studies on housing and elderly health using longitudinal survey data are strongly urged [9,33].

Although it has been widely accepted that housing transcends physical spaces and has social and symbolic significance in the daily life, relationships, and interactions of the elderly [34], research into the participation of the elderly in housing issues is in demand. We know little about how the elderly interact with their living spaces [35], and no consensus has been reached about what makes a housing structure more than just a place of residence. On the one hand, this indicates that the social and economic characteristics of the elderly are important in the correlation between housing and elderly health, and by better understanding family choices and decision-making, the mechanisms of how the elderly participate in housing can be revealed [36]. This may also help clarify the relationship between behavioral and educational interventions and risk prevention in the elderly [37]. Currently, studies within this field have considered limited demographic characteristics such as age, gender, marital status, income or relative income, self-reported economic status, social interaction, and career [23,29]. Including limited socioeconomic factors in a regression may result in missing variables, which can lead to bias in conclusions on the relationship between housing and health.

On the other hand, research into elderly participation in housing needs should also further expand upon housing hazards and mobility obstacles (which have been the focus of housing and health research [19]), to a greater extent involving functions and activities specific to the elderly [38]. Current research has often focused on falls [2], bathing and dressing obstacles [6,19], burns [39], diseases such as Alzheimer’s [40], mental health, and circadian rhythm and sleep quality [26], which are all relevant to the health of the elderly. Investigations of the relationship between housing and activities of daily living (ADLs) and instrumental activities of daily living (IADLs) are important [19].

In this study, to fill the current research gap on the correlation between housing and health, we conducted an index based on an ADL and IADL scale to investigate the influence of housing on the overall abilities of the elderly. We estimated the influence of housing on changes in health status using two-year panel data derived from longitudinal surveys. We also estimated the medical expenditures of the elderly in relation to their health and housing conditions to confirm the importance of housing on the health of the elderly.

## 2. Materials and Methods

### 2.1. Research Design

To explore our research questions, we examined the possible relations between housing and elderly health first through the correlation between housing characteristics and health and second through the dynamic effect of housing on health and on medical expenditures.

#### 2.1.1. The Correlation between Housing and the Health of the Elderly

In the first stage, we analyzed the correlation between housing and the health of the elderly during the survey period. Lots of studies have investigated factors that influence elderly health. The effects of natural attributes, including gender [41], age [42], and nationality [43]; social attributes, including marital status [44], residence [45], education [42], and income [46]; and lifestyle factors, including smoking and drinking [47], exercise, activities [48], disease [48], and mentality [49]; have been examined. In this study, we conducted a health status index as an indicator to describe the overall health of the elderly, including disease and mentality. Thus, in our measurements, disease, mentality, and health status were endogenous. We also included regional factors. Robustness tests are also provided.

We used an OLS regression (Ordinary Least Square regression) in conducting our benchmark study, as shown in Equation (1) below:

(1) $$HFC_{i}=\beta_{0}+\beta_{1}HCC_{i}+\beta_{2}natural\_attributes_{i}+\beta_{3}social\_attributes_{i}+\;\beta_{4}lifestyle\_factors_{i}+\beta_{5}regional\_factors_{i}+\varepsilon_{i}.$$

where *HFC* represents health function credits and *HCC* represents housing character credits. We controlled for natural, social attributes and lifestyle, regional factors. Measuring differences in the elderly who are very healthy is difficult with the ADL/IADL scale, so we further used a Tobit regression model to avoid any possible bias.

#### 2.1.2. The Dynamic Impact of Housing on the Health of the Elderly

In the second stage, we further investigated the impact of housing on dynamic changes in the health status of the elderly. Following Huang [50], we divided health status into three categories: healthy, health-damaged, and functionally disordered.

A state-transition model was used at this stage. The probability that an old person moves from initial health status *i* to status *j* over time *t* is $P_{ij}\left(t\right),i,j=1,2,3$, so we have a state-transition matrix as $P\left(t\right)=\left[P_{ij}\left(t\right)\right]$. Here, 1, 2, and 3 refer to healthy, health-damaged, and functionally disordered, respectively.

$S_{it}$ is a binary variable representing an old person in status *i* at time *t*. An ordered logit model is estimated to capture the role of *HCC* on the probability of switching health statuses between the three states:

(2) $$P(S_{1t}=1|S_{it-1},X)=F\left(\alpha_{1}-\overset{3}{\underset{i=2}{\sum }}\beta_{i-1}S_{it-1}-\beta_{h}HCC-X^{\prime }\beta \right)$$

(3) $$P(S_{2t}=1|S_{it-1},X)=F\left(\alpha_{2}-\overset{3}{\underset{i=2}{\sum }}\beta_{i-1}S_{it-1}-\beta_{h}HCC-X^{\prime }\beta \right)-F\left(\alpha_{1}-\overset{3}{\underset{i=2}{\sum }}\beta_{i-1}S_{it-1}-\beta_{h}HCC-X^{\prime }\beta \right)$$

(4) $$P(S_{3t}=1|S_{it-1},X)=1-F\left(\alpha_{2}-\overset{3}{\underset{i=2}{\sum }}\beta_{i-1}S_{it-1}-\beta_{h}HCC-X^{\prime }\beta \right)$$

where $\alpha_{1}$ and $\alpha_{2}$ are the threshold parameters dividing the health status latent variable into three categories. $\beta_{h}HCC$ and $X^{\prime }\beta$ represent the product of housing characteristics and the control variables with their coefficients.

Using the ordered logit regression model, α and β were estimated for the years 2011 and 2013 by including the control variables of marital status, income, assets, and exercise habits and the interaction between housing conditions and health status. We estimated α and β with an MLE (Maximum Likelihood Estimate). On the basis of the results of α and β, the state-transition model was estimated to capture changes in health conditions in both good and bad housing conditions.

#### 2.1.3. Economic Benefits Analysis: Evidence from Medical Expenditures

For the economic benefits analysis, we first used a regression to estimate the medical expenditures for different health statuses, controlling for age, gender, income, assets, and regional factors. By using the estimated state-transition model, we could estimate the medical expenditures for those living in different housing conditions. By comparing their expenditure growth rate differences, additional medical expenditure growth could be obtained if the elderly were living in poor housing conditions.

### 2.2. Data

Our research was based on the China Health and Retirement Longitudinal Study (CHARLS) from 2011 and 2013, which covered 450 communities in 28 provinces, autonomous regions, or municipalities in China. CHARLS investigated respondents’ demographic characteristics, family structure and support, social interactions, income, consumption, and assets. It also provided detailed information on health, lifestyle, and housing characteristics, which were critical factors in our research. Heads of household who were over 60 years old were identified in the research. After eliminating invalid or missing information from the survey, 4740 and 5437 observations remained from 2011 and 2013, respectively, enabling us to conduct a two-term panel data analysis using 3850 observations.

A statistical description of the variables is presented in Table 1. In the survey, 60% of the heads of household were younger than 70 years old, and fewer than 10% of respondents were over 80.

In this study, we focused on the impact of the physical characteristics of housing on health status. The concept of the “healthy housing environment” [22] identifies housing attributes from the perspectives of safety, health, amenities, and convenience, which is an accepted approach in the literature.

To compare the contributions of different housing characteristics, we standardized all housing characteristic variables as ranging from 0 to 1: the higher the score, the better the housing characteristics for the elderly. It is well known that housing is a bundle of attributes. Thus, in this study, we used housing character credits (HCCs), that is, the sum of the scores of all 13 characteristics of housing, to measure housing conditions: the larger the total score, the better the housing conditions.

We also attempted to use principal components analysis (PCA) to conduct HCCs, but this had two limitations for the current study: based on the 13 housing characteristics, a total of 8 principal components were necessary, and negative terms in the principal components analysis would have been difficult to explain in our study. Thus, we used HCCs in our study. Table 2 presents the descriptive statistics of these housing characteristics.

In the study, the health status of the elderly was measured based on activities of daily living (ADLs) and instrumental activities of daily living (IADLs), which are widely used in studies of elderly health. In the CHARLS, the daily activity ability of elderly people was classified into four levels based on ADLs and IADLs: without assistance, with minor assistance, with extensive assistance, and completely unable to complete without assistance. Three phases were included to investigate ADLs and IADLs in the CHALRS: ADL screening, ADL investigation, and IADL investigation. The ADL screening was designed to distinguish healthy respondents from very unhealthy respondents. Only very unhealthy respondents required an ADL investigation.

To assess the health status of the elderly, we constructed a health function credits (HFC) index. We assigned values of 1, 0.6, 0.3, and 0 to the four levels of ADL screening and IADL investigation. As only the rather unhealthy respondents accepted an ADL investigation, this phase’s points were negatively assigned as 0, −0.03, −0.06, and −0.1 to gain a more accurate picture of the sample. Table 3 presents the HFCs of the different age cohorts. We found that as people got older, their health declined more rapidly and their health status became more dispersed, which reflected the real characteristics of the health status of the elderly.

To simplify the health status description further, we followed Huang [49] and divided the health status of the elderly into the three categories of healthy, health-damaged, and functionally disordered, as shown in Table 4.

## 3. Results

### 3.1. Housing Characteristics and the Health of the Elderly

#### 3.1.1. Basis Regression

Table 5 presents the results for the association of housing with the health of the elderly based on OLS and Tobit regression. HFC was shown to be significantly and positively correlated with HCC. The results were consistent with the two methods. The control variables had the expected signs.

#### 3.1.2. The Heterogeneity Test for Different Age Cohorts

In this section, we further test whether the relationship found above holds for different age cohorts. The heterogeneity test helps us to understand age groups and their relationship to housing and health, which can guide the elderly in determining the best time for adaption and provides implications for effective government subsidies. We divided the respondents into the three age cohorts of 60–70, 70–80, and over 80. The results of the Tobit regression are given in Table 6 for CHARLS 2011. The results for CHARLS 2013 were consistent and thus are not shown here.

The results show that the correlation between housing properties and health was significant for the elderly under 80 years old.

### 3.2. The Dynamic Effect of Housing on Health

Using the two-term panel data, we could track the changes in the health of the elderly. The results of the ordered logit regression are shown in Table 7. The coefficient of HCC also showed that the housing characteristics in 2013 significantly and positively influenced a change in health status. We also added a cross-term for health status in 2011 and housing characteristics, which were $S_{2t-1}HCC$ and $S_{3t-1}HCC$, but the coefficient was not significant. The effect of housing on health status therefore did not depend on the primary health status. The other control variables’ signs were as expected. (There was a possible concern about endogeneity in the relationship between housing and health. We considered and tested the three possible paths that could have caused endogeneity problems to confirm. Please see Appendix A.)

Using status state-transition models (2), (3), and (4) (introduced in Section 2.1), Table 8 shows the estimated results of the different age and housing characteristics. Poor housing conditions refer to those in the bottom 50% of HCC in 2013, while good housing conditions refer to those in the top 50% of HCC from the houses in 2013. The percentages in the matrix indicate the probability of health status changing between 2011 to 2013 in the two housing condition scenarios in the different age groups. For example, for the elderly aged 70–80 in good housing conditions, the housing was either consistently good or improved from a poorer condition in 2011, and the probability of health changing from functionally disordered to health-damaged was 55.75% compared to 49.31% for those in poor housing conditions. 

The left lower triangle of the matrix (in dark gray) shows health improvements, and the right top triangle of the matrix (in white) shows a deterioration. We found that housing improvements positively influenced changes in the health status of the elderly. This influence could differ in terms of age and evolution direction. On average, the positive effect of housing on controlling health deterioration increased from 1.6% to 2.57% with age, but the positive effect on promoting health recovery decreased from 3.92% to 1.20%. The health maintenance impact increased from 4.40% to 7.24%. Thus, housing improvements were more like healthcare products than drugs or treatments.

### 3.3. Economic Benefits Analysis: Evidence from Medical Expenditures

For the elderly, medical expenditures are often a huge burden, particularly for those who are unhealthy. Table 9 shows the estimated medical expenditures in 2013 given the different health statuses. The results show that the medical expenditures of the elderly with impaired health were 44.7% higher than for those who were healthier, and expenditures for those who were functionally disordered were 130.0% higher.

By combining Table 9 and Table 10, we were able to estimate the expenditure growth rate of medical expenditures for the elderly living in poor housing conditions in 2013 compared to those living in good housing conditions at different ages. Here, we define RDGR (relative difference of growth rate) to describe the relative difference growth rate of medical expenditures. Table 10 shows that the growth of medical expenditures was accelerated for those elderly people living in poor housing conditions. The housing conditions of the healthy elderly of all ages had greater economic benefits than for those in health-damaged and functionally disordered conditions, and the benefits declined with age. These results suggest that housing adaptation is more important in terms of economic benefits for younger and healthier older people. Thus, government housing adjustment subsidies for such elderly people will be more efficient.

## 4. Discussion and Conclusions

The effect of housing on the health of elderly has been widely studied. However, as discussed, the relationship between housing and health is complex because of the interaction between the competence of the elderly and housing. To assess the impact of housing conditions on the independence and functional capabilities of the elderly, two indices were built in this study to comprehensively examine housing characteristics and the health of the elderly. The relationship between these factors and the dynamic impact of housing improvements on changes in the health of the elderly were measured. In addition, the economic benefits of housing improvements were examined from the perspective of medical expenditure savings. Our study confirmed previous research indicating that the condition of housing has a positive relationship with the function and health status of the elderly. We also demonstrated that housing improvements can have actual benefits on the health of the elderly and on their medical spending.

The health of the elderly continues to be one of the most important global public health issues. Although the empirical tests in this paper were based on Chinese data, our methods and conclusions are also valuable for other countries facing pension challenges. Our study first provides evidence of the importance of healthy housing adaptation for the elderly and provides a new direction for public health policies through their integration with housing policies. Housing adaptation is a challenge for the elderly not only because of the construction process but also because of the financial burden, particularly for those on low incomes or who are older. Housing policies that aim to support the aging population can therefore affect housing affordability and public health. Our estimates of the economic benefits of housing improvements for the elderly indicate that efficient subsidized policies should target younger and healthier older people. Thus, education about the importance of age-friendly housing to health and the economy at an earlier and healthier age is therefore necessary.

Our results suggest that housing as a whole is more important for the health of the elderly than any single attribute, although this was not the focus of our study. Further research is required to fully understand this mechanism. However, our results do reflect the characteristics of housing as a bundle of attributes and imply that aging-friendly housing requires systematic planning and design.

Due to the limitations of the data, we only estimate the short-term effect of housing on the health of the elderly and only consider the cost of medical expenditures. Further studies can address the long-term effects of housing on health and include other costs, such as those for formal or informal services for unhealthy elderly people. When data are available, future empirical tests should be extended to more markets and international comparisons should be made to better understand the relationship between housing and the health of the elderly.

## Figures and Tables

**Table 1:** Descriptive statistics of the elderly’s demographic variables.

Variable	Description	Mean	SD
		Year 2013	
*Age*	The age of the household head	69.27	7.72
*Female*	1 if female, 0 if male	0.47	-
*Urban*	1 if urban community, 0 if other	0.39	-
*East*	1 if eastern region in China, 0 if other	0.31	-
*Minority*	1 if national minority, 0 if other	0.07	-
*Marriwith*	1 if married and living with spouse, 0 if other	0.63	-
*logWEALTH_PC*	The total wealth per capita (RMB) of the household	10.31	1.46
*logINCOME_PC*	The annual income per capita (RMB) of the household	8.52	1.34
*Exercise*	Times of activity above 10 min per week	0.29	0.45
*Smoking*	1 if previously smoked, 0 if other	0.48	-
*Drinking*	1 if drinks more than once per month, 0 if other	0.12	-

Note: (1) log is a natural logarithm. Data source: China Health and Retirement Longitudinal Study (CHARLS) 2011 and CHARLS 2013.

**Table 2:** Descriptive statistics of housing characteristics.

Variable	Description	Mean	SD
		Year 2013	
*Hstructure*	1 if reinforced concrete structure, 0 if other	0.34	-
*Hage*	building age, with more credits for new buildings	0.21	0.15
*Htype*	Housing type: 1 if buildings and single bungalows, 0 if other	0.98	-
*Hstorey*	Building story, with more credits for a lower story	0.88	0.25
*Hbarrierfree-steps*	Barrier-free passageway and steps needed, with more credits for fewer steps or barrier-free passageways	0.88	0.26
*Hrooms*	Number of rooms, with more credits for more rooms	0.764	0.15
*Htoilets*	Number of toilets: 1 if more than 2 toilets, 0.8 if 1 toilet, 0 for no toilet	0.63	0.37
*Htoiletstype*	1 if seated toilet, 0 if other	0.21	-
*Helec*	1 if electrical supply available, 0 if not	0.99	-
*Hwater*	1 if water supply available, 0 if not	0.70	-
*Hbath*	1 if bathing facilities available, 0 if not	0.42	-
*Htidy*	Interviewer’s subjective judgment of housing tidiness	0.37	0.21
*HCC*	Sum of all 13 credits above	7.34	1.49

Note: All variables were standardized on a 0–1 scale: the higher the score, the better the housing characteristics for the elderly.

**Table 3:** HFCs in different age cohorts (data source: CHARLS 2011 and 2013).

Variable	Sample	Mean			SD		
		60–70	70–80	Over 80	60–70	70–80	Over 80
*HFC*	2013	10.67	9.86	8.56	1.72	2.42	3.24
	2011	10.76	10.13	8.24	1.71	2.33	3.44

Note: HFC is the abbreviation for health function credits. We assigned values of 1, 0.6, 0.3, and 0 to the four levels of activities of daily living (ADL) screening and instrumental activities of daily living (IADL) investigation and negatively assigned 0, −0.03, −0.06, and −0.1 to ADL investigation. We conducted an HFC index by adding the credits together: the higher the HFC, the healthier the elderly people were.

**Table 4:** Health status division principle.

Health Status	Standard
Healthy ($S_{1}$)	No ADL or IADL disorders
Health-damaged ($S_{2})$	One or more IADL disorders or 1–2 ADL disorders
Functionally disordered ($S_{3})$	More than 3 ADL disorders

Note: “disorders” refers to respondents who need extensive levels of assistance for a specific ADL/IADL term, the third and fourth levels of the ADL/IADL scale.

**Table 5:** Correlation between housing and health status (benchmark and Tobit regression).

	(1)	(2)	(3)	(4)
Year	2011		2013	
	OLS	Tobit	OLS	Tobit
*HCC*	0.0847	0.115	0.0977	0.127
	(0.00) ***	(0.00) ***	(0.00) ***	(0.00) ***
*age*	−0.103	−0.121	−0.0889	−0.104
	(0.00) ***	(0.00) ***	(0.00) ***	(0.00) ***
*female*	−0.710	−0.997	−0.731	−1.036
	(0.00) ***	(0.00) ***	(0.00) ***	(0.00) ***
*Urban*	0.0684	0.101	0.00303	0.00630
	(0.35)	(0.25)	(0.96)	(0.94)
*East*	0.0722	0.102	0.267	0.350
	(0.33)	(0.25)	(0.00) ***	(0.00) ***
*Marriwith*	0.231	0.248	0.243	0.247
	(0.00) **	(0.00) **	(0.00) ***	(0.00) **
*logWEALTH_PC*	0.0420	0.0744	0.0666	0.0976
	(0.08)	(0.01) **	(0.00) **	(0.00) ***
*logINCOME_PC*	0.135	0.173	0.248	0.307
	(0.00) ***	(0.00) ***	(0.00) ***	(0.00) ***
*Exercise*	0.429	0.446	0.410	0.465
	(0.00) ***	(0.00) ***	(0.00) ***	(0.00) ***
*Smoking*	−0.0459	−0.0911	−0.0810	−0.126
	(0.58)	(0.36)	(0.31)	(0.18)
*Drinking*	−0.194	−0.277	−0.345	−0.459
	(0.10)	(0.05) *	(0.00) ***	(0.00) ***
*_cons*	15.38	16.29	12.86	13.34
	(0.00) ***	(0.00) ***	(0.00) ***	(0.00) ***
*sigma*				
*_cons*		2.441		2.396
		(0.00) ***		(0.00) ***
*N*	4253	4253	5030	5030

Notes: HCC is the abbreviation for housing condition credits, which is the sum of all 13 housing character indices. The dependent variable was HFC, which was used by the authors to measure the health status of the elderly. The *t*-statistic is in parentheses; * *p* < 0.05; ** *p* < 0.01; *** *p* < 0.001.

**Table 6:** Relationship between housing and health status in different age cohorts for CHARLS 2011.

	(1)	(2)	(3)
	Age 60–70	Age 70–80	Over 80
*HCC*	0.104	0.176	0.00751
	(0.00) **	(0.00) ***	(0.94)
*Control Variables*	*Y*	*Y*	*Y*
*_cons*	12.49	18.00	20.46
	(0.00) ***	(0.00) ***	(0.00) ***
*sigma*			
*_cons*	2.000	2.364	3.304
	(0.00) ***	(0.00) ***	(0.00) ***
*N*	2049	1584	620

Notes: Regressions (1), (2), and (3) are subsample Tobit regressions by age. The control variables include age, gender, marital status, income, assets, regional factors, and lifestyle, as specified in Table 5. The control variables had the expected signs. The *t*-statistic is in parentheses; ** *p* < 0.01; *** *p* < 0.001.

**Table 7:** Ordered regression results from the dynamic impact analysis.

	(1)	(2)
	Health Status 2013	Health Status 2013
$HCC_{2013}$	−0.0899	
	(−2.27) *	
$HCC_{2011}$		−0.0637
		(−1.49)
$S_{2,2011}\times HCC_{2013}$	0.112	
	(1.67)	
$S_{3,2011}\times HCC_{2013}$	−0.00232	
	(−0.01)	
$S_{2,2011}\times HCC_{2011}$		0.0619
		(0.88)
$S_{3,2011}\times HCC_{2011}$		0.174
		(0.91)
$S_{2,2011}$	0.772	1.136
	(1.87)	(2.76) **
$S_{3,2011}$	4.022	3.047
	(3.31) ***	(2.71) **
*Control Variables*	*Y*	*Y*
$\alpha_{1}$		
*_cons*	3.432	4.357
	(5.26) ***	(6.93) ***
$\alpha_{2}$		
*_cons*	6.434	7.342
	(9.63) ***	(11.35) ***
*N*	3642	3476

Notes: Regression (1) uses HCC in 2013 and control variables in 2013. Regression (2) uses HCC in 2011 and control variables in 2011. Health status is divided into the three categories of healthy ($S_{1}$), health-damaged ($S_{2}$), and functionally disordered ($S_{3})$. $S_{i,t}$ means health status is in the $S_{i}$ level at time *t*. For example, $S_{3,2011}$ = 1 means the household was functionally disordered in 2011. The control variables include age, gender, marital status, income, assets, regional factors, and lifestyle, as specified in Table 5. The *t*-statistic is in parentheses; * *p* < 0.05; ** *p* < 0.01; *** *p* < 0.001.

**Table 8:** Health status transition matrix by age and housing.

Health Status 2011	Good Housing Condition in 2013			Poor Housing Condition in 2013		
	Health Status 2013			Health Status 2013		
	$S_{1,2013}$	$S_{2,2013}$	$S_{3,2013}$	$S_{1,2013}$	$S_{2,2013}$	$S_{3,2013}$
	Years 60–70			Years 60–70		
Healthy ($S_{1,2011})$	90.13%	9.33%	0.54%	85.73%	13.45%	0.82%
Health damaged ($S_{2,2011})$	64.06%	33.23%	2.71%	60.68%	36.20%	3.12%
Functional disordered ($S_{3,2011})$	14.28%	62.74%	22.98%	9.82%	58.85%	31.33%
	Years 70–80			Years 70–80		
Healthy ($S_{1,2011})$	82.76%	16.21%	1.02%	77.04%	21.50%	1.46%
Health damaged ($S_{2,2011})$	48.78%	46.26%	4.96%	46.51%	48.09%	5.41%
Functional disordered ($S_{3,2011})$	8.05%	55.75%	36.20%	5.74%	49.31%	44.95%
	Years over 80			Years over 80		
Healthy ($S_{1,2011})$	71.18%	26.85%	1.97%	63.94%	33.33%	2.73%
Health damaged ($S_{2,2011})$	32.99%	57.84%	9.17%	31.79%	58.58%	9.63%
Functional disordered ($S_{3,2011})$	4.31%	43.23%	52.46%	3.11%	36.17%	60.72%

Notes: (1) Good housing conditions refer to those in the top 50% of HCC in 2013, while poor housing conditions refer to those in the bottom 50% of HCC from houses in 2013. (2) The numbers in the matrix indicate the probability that health status changed between 2011 and 2013 in the two scenarios of housing conditions in the different age groups. The left lower triangle of the matrix (in dark gray) shows health improvements, and the right upper triangle of the matrix (in white) shows health deterioration. The diagonal reflects that the present status was maintained (in light gray).

**Table 9:** OLS regression results for medical expenditures.

	(1)
	*logMedical*
	2013
Health-damaged	0.447
	(5.91) ***
Functionally disordered	1.300
	(5.53) ***
*Age*	0.00512
	(1.01)
*Female*	0.237
	(3.75) ***
*Urban*	0.139
	(2.08) *
*East*	0.208
	(3.17) **
*Marriwith*	0.273
	(4.01) ***
*logINCOME_PC*	0.219
	(7.85) ***
*logWEALTH_PC*	0.00129
	(0.06)
*_cons*	3.615
	(7.67) ***
*N*	2783

Notes: Regression (1) uses cross-sectional data from 2013. The regression results with cross-sectional data in 2011 had the same sign. Medical expenditures are treated with a logarithm. The *t*-statistic is in parentheses; * *p* < 0.05; ** *p* < 0.01; *** *p* < 0.001.

**Table 10:** Additional medical expenditures of those in poor housing conditions.

Health Status in 2011	RDGR	Average RDGR
Years 60–70		
$S_{1,2011}$ healthy	45.27%	23.71%
$S_{2,2011}$ health-damaged	10.12%	
$S_{3,2011}$ functionally disordered	15.74%	
Years 70–80		
$S_{1,2011}$ healthy	34.42%	17.14%
$S_{2,2011}$ health-damaged	5.21%	
$S_{3,2011}$ functionally disordered	11.80%	
Years over 80		
$S_{1,2011}$ healthy	26.67%	12.60%
$S_{2,2011}$ health-damaged	2.46%	
$S_{3,2011}$ functionally disordered	8.66%	

Note: The percentage is the additional medical expenditures of the elderly in 2013 living in poor housing conditions compared to those in good housing conditions at different ages. RDGR: relative difference of growth rate.

## Appendix A. A Further Discussion of Endogenous Problems

In this study, we focused on measuring the role of housing in the health of the elderly, but there was a possible concern about endogeneity in the relationship between housing and health, which may have biased the findings. To confirm our results, we considered and tested the three possible paths that could have caused endogeneity problems. Due to space limitations, we do not show the results, but they are available from the authors upon request.

First, there may be an adverse selection relationship between housing and health, which means that only those who maintain a certain level of health are likely to live in relatively poor housing conditions. Thus, if their health deteriorates, they may require some improvements to their housing to meet their survival needs. Second, the healthier elderly, who typically have lower levels of medical expenditures, may be more likely to use their savings for home modifications, while others may need to maintain more of their savings as a precaution. Third, health status may influence elderly people’s ability to do housework, which can influence HCC through the tidiness of their housing. We thus tested all three possible causes of endogeneity between housing and health. Our results confirmed the reliability of all of our findings.
